# Patient's Decision to Disclose the Use of Traditional and Complementary Medicine to Medical Doctor: A Descriptive Phenomenology Study

**DOI:** 10.1155/2018/4735234

**Published:** 2018-02-14

**Authors:** Johny Anak Kelak, Whye Lian Cheah, Razitasham Safii

**Affiliations:** Department of Community Medicine and Public Health, Faculty of Medicine and Health Sciences, University Malaysia Sarawak, Sarawak, Malaysia

## Abstract

Nondisclosure of traditional and complementary medicine (T&CM) use may cause individual to be at risk of undue harm. This study aimed to explore patient's experience and views on their decision to disclose the use of T&CM to the doctor. An exploratory qualitative study using in-depth interview involving 10 primary care clinics attendees in Kuching was conducted. The results indicated that disclosure of T&CM use will motivate them to get information, increase doctor's awareness, and get support from family and friends for disclosure. Fear of negative relationship and negative response from doctors was a barrier for disclosure. Doctor's interpersonal and communication skills of being involved, treating patients respectfully, listening attentively, respecting privacy, and taking time for the patient were a critical component for disclosure. Intrapersonal trust regarding doctor influences their satisfaction on healthcare. Women are more open and receptive to a health concern and expressing negative emotions and tend to share problems, whereas men always described themselves as healthy, tended to keep their own personal feeling to themselves, and tended to not share. The doctor should consider gender differences in disclosure, their attitude towards T&CM use, and gained patient's trust in the delivery of healthcare services. Good interpersonal and communication skills must be maintained between doctor and patients.

## 1. Introduction

Ensuring safety, efficacy, and quality of traditional and complementary medicine (T&CM) products is a core objective of World Health Organization (WHO) towards recognizing the role of T&CM in modern healthcare system [1]. However, a major issue of importance involving the coordination of T&CM and conventional medicine is whether or not T&CM users disclose their use of T&CM to their healthcare providers and the nondisclosure of use of T&CM may cause individual at risk of undue harm [2, 3].

The review of the existing literature on T&CM disclosure estimates was relatively low globally (between 7.6% and 48.2%) and Malaysian studies showed almost similar scenario in which the nondisclosure rate was as low as 25% and as high as 90.4% [4–10]. Hence, this lack of discussion may indicate a deficiency in patient-conventional healthcare professional relations and could have a negative impact on patient care and outcome [11]. In particular, traditional and complementary medicine is thought by many people to be natural and safe to use, without adverse effects. Most people use traditional and complementary medicine in addition to conventional medicine.

In Malaysia, particularly in multiethnic-multicultural areas like Sarawak, though many studies have investigated the determinants of T&CM use as well as the decision to disclose the T&CM use, few have explored in depth the factors that determine the disclosure of T&CM use to the medical doctor. In continuation of our quantitative studies on the determinant of T&CM disclosure, this qualitative study was conducted in order to explore participants' views in more depth and provide additional insight [10]. Thus, this study aimed to explore the experience and views of primary care clinic attendees in Kuching Division, Sarawak, in relation to their decision to disclose the use of T&CM to the medical doctor.

## 2. Materials and Methods

### 2.1. Study Design

A descriptive phenomenology study based on semistructured guided interviews with participants was conducted to understand their experience and views on disclosure of T&CM use to the medical doctor. The interview was undertaken between January and April 2017. Thematic analysis approach was used in this present study as it offers a flexible approach to analyzing qualitative data in identifying, analyzing, and discovering the themes [12].

### 2.2. Settings, Sampling, and Participants

The study was conducted in three selected primary healthcare clinics in Kuching Division, Sarawak. Sarawak is the largest state in Malaysia, with a population of approximately 2.8 million and it is the least densely populated state among 13 states in Malaysia. Kuching Division is one of the eleven administrative divisions in Sarawak and it consists of three administrative districts: Kuching, Bau, and Lundu. Healthcare in Sarawak is, administratively under Sarawak Health Department, delivered through a network of static and mobile facilities that provide promotive, preventive, curative, and rehabilitative care. In this study, three primary healthcare clinics were identified as the research sites for the logistics and because of the fact that within these three healthcare clinics the highest numbers of the attendance rates and medical officers exist [13]. All interviews were conducted privately in the doctor's consultation room that was provided by the health clinic management.

Participants who fit the inclusion criteria for selection of participants which were being Malaysian, being 18 years of age or older, using at least one type of T&CM [14] for the past 12 months, and attending outpatient department of three selected primary healthcare clinics were approached prior to this study in our pervious quantitative study [10]. Those participants who had agreed to participate in this current study during the previous study [10] were selected via purposive sampling. The recruitment of participants was continued until the saturation point was reached at 10th interview and no new information was obtained from the subsequent interviews [15].

### 2.3. Interview Tool

A semistructured interview guide was used for the purpose of data collection. The interview guide was developed based on the findings of our previous study [10], where the list of possible questions to assess the participant's views on T&CM and status of disclosure was identified. It consists of open-ended questions, such as “tell me your experiences about the disclosure of the use of traditional and complementary medicine to doctor?”, so that it can provide interviewees with maximum opportunity to express their views. Interview guide questions were evaluated among research team in terms of its relevance and appropriateness. These were translated into Bahasa Malaysia, which could be understood by the majority of the participants. A bilingual language expert was asked to verify the translation from English to Bahasa Malaysia for its accuracy and for appropriateness of the words used in the translated version* (see Appendix)*.

### 2.4. Data Collection

Data collection was performed using one-to-one in-depth interviews of participants. Before the interviews, the purpose and process of the study were explained to the participant by the researcher. The participants read through the participant information sheets and the researcher encouraged the participant to raise questions about the study, which were answered accordingly. Verbal consent as well as written informed consent was obtained from participant. All interviews were audio-recorded and the researcher attended all interviews while the research assistant took some of the field notes. Each interview lasted for approximately 15 to 20 minutes. The interviews were in Malay language and were mainly focused on study objectives, and probing questions were used when necessary to get a more thorough understanding of the issue involved. Participant's demographics and health profile were also obtained prior to interviews. The content of the interviews was considered to be saturated when no new information was obtained from participants. The interview transcripts were not returned to participants for comment but the researcher engaged participants immediately following the interview and no addition or correction was made. The interviews were transcribed verbatim.

### 2.5. Data Analysis

The audio-recorded interview in the Malay language was transcribed and translated into English by the researcher. All transcribed interviews were later subjected to thematic analysis and the transcripts were analyzed manually to identify the emerging themes [12]. Steps in the thematic analysis included: (1) being familiarised with data by reading through the transcript, line-by-line; (2) generating initial codes; (3) searching for themes by aggregating similar codes together; (4) reviewing themes by connecting and interrelating themes; (5) defining and naming themes; and (6) producing the report. Throughout the steps, findings were constantly discussed by research team.

### 2.6. Study Rigour

To establish the rigour of this study, researcher considered the credibility, dependability, and transferability of each participant's interview [16]. To ensure the credibility, audit trail was followed throughout the data collection to make sure researcher interpreted what participant reported correctly. Selection of the most suitable codes and themes was also ensured by discussion among members. To ensure dependability of this study, the same interview guide was used with all participants to ensure consistency during data collection. Proper selection of participants, data collection, and process analysis with appropriate quotations will allow readers to judge transferability of these findings.

### 2.7. Ethical Consideration

The ethical approval was obtained from the Ethics Committees from the University Malaysia Sarawak (UNIMAS) and National Medical Research Register (NMRR) Ministry of Health Malaysia (NMMR-16-178-29371). Verbal and written consent were obtained from those who agreed to participate in this study and their anonymity, confidentiality, and freedom to leave the study were assured.

## 3. Results

### 3.1. Description of Study Participants

A total of ten participants with a mean age of 31.6 (SD = 6.6) years were interviewed for data generation. The age of participants ranged from 20 to 40 years, with the majority of them being in the age group of 31 to 40 years (60%), 21 to 30 years (30%), and less than 20 years (10%). The majority of the participants interviewed were female (*n* = 7) and only 3 of them were male. The participants are comprised of different ethnic groups, namely, the Malays (40%), Chinese (10%), and Bidayuh (60%). In terms of religion, half of them were Christians (50%) and 40% of them were Muslims, whereas the other 10% were Buddhists. The majority of the participants were married (80%). All participants received formal education with half of them receiving formal education till the tertiary school level (50%) and 40% of them till secondary school level, whereas the other 10% received formal education till primary school level. Among the participants, 50% of them reported a monthly household income of MYR1000 and below and the other 10% reported the household income of MYR2000 and above. About 40% of them were unemployed with two of them being housewives, and the rest were students (*n* = 1) and a farmer (*n* = 1). Among those who were working, 30% were in private (nongovernment) sectors and similarly 30% were in government sectors. All participants had at least one medically diagnosed chronic diseases, where half the participants had been diagnosed to have diabetes mellitus (50%) and hypercholesterolemia (50%), followed by hypertension (40%) and asthma (30%). All participants reported using supplement within previous 12 months. Only four participants reported using multiple types of traditional and complementary medicine in which two of them had tried body massage (*n* = 2) and the rest used Malay herbs (*n* = 1) and acupuncture (*n* = 1) for their illness (Table 1).

### 3.2. Themes

**Figure 1:**
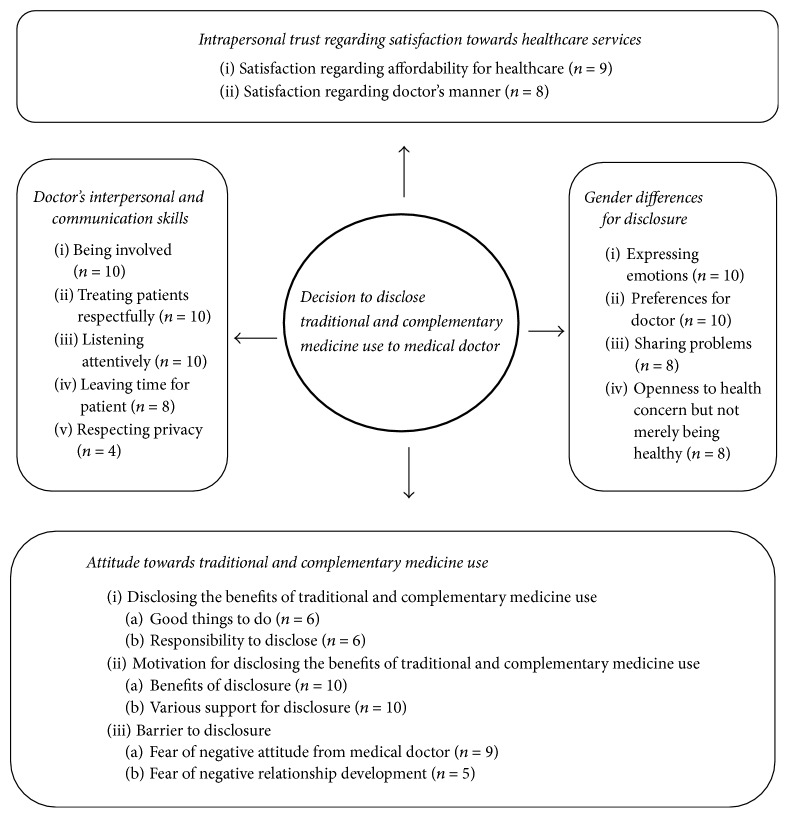
Summary of the factors that influence participants to disclose the use of traditional and complementary medicine to medical doctor.* Notes*. Circle/rectangle: salient themes/subthemes. Numbers: frequency.

Four key themes contributed to the experiences and views of the participants in relation to their decision to disclose the use of T&CM to medical doctor: (1)* attitude towards traditional and complementary medicine use*; (2)* doctor's interpersonal and communication skills*; (3)* intrapersonal trust regarding satisfaction towards healthcare services*; and (4)* gender differences regarding disclosure* (Figure 1). The participants did not provide feedback on the findings. These are outlined below.

#### 3.2.1. Attitude towards Traditional and Complementary Medicine Use

Participants experienced both positive and negative attitude towards traditional and complementary medicine. Although the participants expressed the negative attitude towards the barrier to these practices, the majority of them claimed that disclosing the benefits of these practices could motivate them more to use the traditional and complementary medicine after disclosing them to their doctor.

*Disclosing the Benefits of Traditional and Complementary Medicine Use*. Some of the participants perceived that disclosing the benefits of T&CM use was a good thing to do and that it was their responsibility to disclose it to their doctor, which could be illustrated by the following excerpts:

…For me it is good to inform your doctor, so that they will be aware that we use this medicine. At least they can monitor our health. They can give some advice if any problem occurs due to this medicine…. (*Participant*  10/♀/*Malay*/25 *yrs*/*Teacher*)

…I am curious about a certain supplement which is proven can improve some of the chronic diseases. I think it is important to consult my doctor regarding of this issues, so that I am confident to try this medicine. I want assurance from the doctor…. (*Participant*  6/♂/*Malay*/40 *yrs*/*Farmer*)

…As a patient, it is my responsibilities to inform the doctor of what I have especially on the medicine. I am concern of my health and afraid if I take the wrong decision without consulting my doctor first. Although this traditional medicine has many benefits, I think I still need to consult my doctor because I want to get the correct information of this medicine…. (*Participant*  1/♀/*Bidayuh*/35 *yrs*/*Teacher*)

*Motivators for Disclosing the Benefits of Traditional and Complementary Medicine Use*. All participants perceived that what motivates them to disclose their use of traditional and complementary medicine to the medical doctor was a range of potential benefits of the doctor knowing of their patient use of these practices. The main benefits of disclosing it to their medical doctor included health benefits and medium of decision-making which could be illustrated by the following excerpts:

…I think the doctor should know about traditional medicine. I believed it can improve our sickness through more gentle way without introduction of pill which may contain many chemicals that may harm to our body. I hope that the doctor knows about the benefits of this medicine…. (*Participant*  2/♀/*Bidayuh*/37 *yrs*/*Promoter*)

…Besides that, I want more explanation of traditional medicine from the doctor as I am curious about the effectiveness of this medicine in improving our sickness. After all, the doctor should know about this traditional medicine, so that they can decide for us, which one is better for our health either continue to use it or not…. (*Participant*  3/♂/*Bidayuh*/30 *yrs*/*Waiter*)

Moral support from their spouse, family, and friends encourages participants to disclose the use of T&CM especially regarding the advantages and disadvantages of T&CM to the medical doctor which could be illustrated by the following excerpts:

…Well. My family and husband fully support for me to use this medicine. They are also using this supplement. They encourage me to tell doctors if any problem happens to get reassurance form doctor…. (*Participant*  1/♀/*Bidayuh*/35 *yrs*/*Teacher*)

…Well. My wife also uses this supplement. She always asking me on her behalf to consult a doctor about the advantages and disadvantages of this supplement…. (*Participant*  6/♂/*Malay*/40 *yrs*/*Farmer*)

…Well. A friend of mine always gives their encouragement for me to use this supplement. They believe this supplement is safe and have a lot of benefits, which I also agree with that. They also don't mind for me to consult a doctor about this supplement, just for reassurance…. (*Participant*  2/♀/*Bidayuh*/37 *yrs*/*Promoter*)

*Barrier to Disclosure*. Most of their reasons for avoiding disclosure to their doctor were influenced by doctor's behaviours which discouraged them from disclosing their T&CM use, which could be illustrated by the following excerpt:

…Well. Some doctor might not so supportive to explain further about this traditional medicine. They know that this medicine might not sure of its safety and effectiveness. They are not encouraging their patient to consume this medicine…. (*Participant*  8/♂/*Bidayuh*/20 *yrs*/*Student*)

Furthermore, most of the participants feared their doctor's negative emotions, such as anger, and being scolded if they continued asking about traditional and complementary medicine.

…Well. I don't think I ever ask the doctor to help me to choose which traditional medicine is good for me. I am worried that doctor might angry and scold me if I ask him to convince him to choose traditional medicine instead of western medicine…. (*Participant*  6/♂/*Malay*/40 *yrs*/*Farmer*)

Some of the participants stated that a barrier to disclosing the use of traditional and complementary medicine was being afraid that if they disclose the use of these practices, the level of the trust between doctor and participants will be affected, which could be illustrated by the following excerpt:

…and doctor will think that I do not listen to the advice of doctors by taking traditional medicines. So I worried, next time I see him, he would not believe me anymore…. (*Participant*  2/♀/*Bidayuh*/37 *yrs*/*Promoter*)

#### 3.2.2. Doctor's Interpersonal and Communication Skills

All ten participants highlighted that their decision to disclose traditional and complementary medicine use was influenced by doctor's interpersonal and communication characteristics. They perceived that their doctor was considered to be involved in their interaction if their doctor showed sincere involvement and adopted a personal approach in their routine consultations, which could be illustrated by the following excerpts:

…Furthermore, some doctors are very passionate about their patient, as they are always concern and ask their patient's problem…. (*Participant*  3/♂/*Bidayuh*/30 *yrs*/*Waiter*)

…there are no problems for me to disclose the use of traditional medicines. They always ask my problem and opinion related to my illness as well as medication that I take, every time I see them…. (*Participant*  2/♀/*Bidayuh*/37 *yrs*/*Promoter*)

Most of them described their doctor being polite and friendly, which could be illustrated by the following excerpt:

…Like me, the doctor always starts with a good conversation, talk softly and politely and greet his patient and give me to talk until I finish, and they always keep the eye on what I'm trying to say to them…. (*Participant*  9/♀/*Bidayuh*/35 *yrs*/*Housewife*)

Most of the participants believed that doctors were paying full attention during the conversation with good eye contact, which could be illustrated by the following excerpt:

…They have a good eye contact and listener when the conversation with me. I don't feel so awkward when talking with my doctor. The doctor never cut me off when I'm talking and express my problem to them and able to explain appropriately…. (*Participant*  2/♀/*Bidayuh*/37 *yrs*/*Promoter*)

They believed that some of the doctors have good interpersonal skills in their conversation as they are always leaving time for their patient to explain their problems, which could be illustrated by the following excerpt:

…Furthermore, the doctor so far never in rush mood when they talk to me. They always trying whenever possible to satisfy me, and explaining my query. With that attitude, I think not a problem for me to disclose to them…. (*Participant*  4/♀/*Chinese*/25 *yrs*/*Clerk*)

They believed that the doctors dealt with their conversations correctly with confidentiality and keeping privacy, which could be illustrated by the following excerpt:

…they are always kept their patient in private mood during the conversation. They even ask my permission whether her nurse can stay in the same room, when we talking about sensitive issues like female problems…. (*Participant*  1/♀/*Bidayuh*/35 *yrs*/*Teacher*)

#### 3.2.3. Intrapersonal Trust regarding Satisfaction towards Healthcare Services

Most of the participants could utilize the trust built in the relationship with their doctor to facilitate their satisfaction towards healthcare services in order to help them be more closer to the doctor, hence encouraging them to disclose the use of T&CM. They perceived that doctors who give high quality of services such as professionally and credibility in treatment, make them more satisfied with the healthcare services that they received, which could be illustrated by the following excerpt:

…For me it [financial] does not prevent me to receive healthcare services. I can spend more money to get healthcare service in private. For me, government clinic also not a problem for me to access and receive healthcare services. I more satisfy here as I can get free healthcare services here…. (*Participant*  1/♀/*Bidayuh*/35 *yrs*/*Teacher*)

…The issue of expenses is not a problem for me to disclose the use of this medicine to doctors. I prefer the clinic without particularly incur too much on expenses. This is because I will able to go to the clinic without any problem if I need a doctor. If doctor shows their credibility to treat their patient, and act professionally, it can build trust between patient and doctor, I think not a problem for me to share or tell them…. (*Participant*  2/♀/*Bidayuh*/37 *yrs*/*Promoter*)

Most of the participants put their trust in doctors that have good manners and are friendly during conversation which make them more satisfied with the healthcare services that they received, which could be illustrated by the following excerpt:

…Doctors are very friendly and our relationship was good. I feel very confident and not afraid and ashamed to share the problem with the doctor. I trusted the doctor in this clinic. If I trusted the doctor, I know they can do better and act professionally when treating their patient. So I feel more comfortable with that and more satisfaction with their services…. (*Participant*  5/♀/*Malay*/31 *yrs*/*Housewife*)

#### 3.2.4. Gender Differences regarding Disclosure

There were gender differences in health-seeking behaviour and ways of communication. Women are more open and receptive to a health concern and expressing negative emotions and tend to share problems, which could be illustrated by the following excerpts:

…It is certain to get a doctor's opinion on this medicine. The side effects of this medicine, functionality and content of this medicine. I was afraid if over eating and would be harmed to my health…. (*Participant*  4/♀/*Chinese*/25 *yrs*/*Clerk*)

…Nothing to hide from the doctor, as you may explain and tell your doctor anything that you are not sure. Sharing your problem that you are not sure about that, is the best way to avoid any unnecessarily bad happen to you. At least doctor know what we take for our medications…. (*Participant*  2/♀/*Bidayuh*/37 *yrs*/*Promoter*)

…It is important to tell the doctor about our problem and does not hide from the doctor when it comes to health issues although we think that we are fit to be healthy…. (*Participant*  5/♀/*Malay*/31 *yrs*/*Housewife*)

Men always describe themselves as healthy, tend to keep their own personal feelings to themselves, and tend to not share, which could be illustrated by the following excerpts:

…sometimes, I feel nothing happen if we do not tell anything or hide from our doctor. I believe that T&CM is from natural not form chemical things, so it kind of safe thing to eat…. (*Participant*  6/♂/*Malay*/40 *yrs*/*Farmer*)

…Sometimes I'm reluctant to share all my personal issues to the doctor. I think not necessarily to share everything as we can get it personally from Internet…. (*Participant*  6/♂/*Malay*/40 *yrs*/*Farmer*)

…Unlike male, I think they are not too bothered about this supplement. I mean, male not to worries about the side effect of this supplement. As long as it safe, they would not tell anybody. They think they look healthy, so that they don't bother to know and telling the doctor about this supplement…. (*Participant*  2/♀/*Bidayuh*/37 *yrs*/*Promoter*)

However, both preferred* doctors of the same gender* for disclosure, which could be illustrated by the following excerpts:

…Of course it is better for us to share our problem with the same gender. There are more appropriate and we will not shy to share our problem in detail to a male doctor as compare to female doctor…. (*Participant*  6/♂/*Malay*/40 *yrs*/*Farmer*)

## 4. Discussion

This study aimed to explore the experiences and views of primary care clinic attendees in relation to their decision to disclose the use of T&CM to the medical doctor. This study identified some important factors that influenced their decision to disclose. That is, their attitude towards T&CM use, doctor's interpersonal and communication skills, intrapersonal trust regarding satisfaction towards healthcare services, and gender differences regarding disclosure.

No doubt, this study revealed that effective doctor-patient discussion on T&CM is important in order to reduce the danger of T&CM-drug interactions [17]. In this study, we found that several themes cited by participants evidenced some degree of conceptual overlap with one another. For instance, doctor's behaviours, along with participants' behaviour such as emotional reactions and intrapersonal trust regarding the doctor, might affect the quality of the doctor-patient relationship, which was reported to influence disclosure [18].

Our findings showed that, despite of different attitude towards T&CM use, most of the participants felt that disclosure to the medical doctor is important and a good thing to do. It is their responsibility to tell their doctors and, in return, doctors will know the T&CM that they use and learn more about T&CM. Moreover, it will make them more confident to use T&CM after reassurance given by their doctor about T&CM. This finding was similar to the Malaysian study on cancer patients as they believed that disclosing T&CM use with doctors is important as they know better what to take and what not to take [19]. However, as stated from the previous study, there was a lack of knowledge of the benefits, harms, and evidence for herbal medicine in physicians [20]. Furthermore, patient is also reluctant to reveal their usage due to the perception that physician is not knowledgeable of these practices or will disapprove of alternative treatment [21]. This can be explained by the fact that doctors throughout their medical career only learn about modern therapies and seldom learn about traditional medicine, hence the lack of doctor's interest in these practices [19]. Some of the participants were often preoccupied with a feeling of this responsibility and desired to tell their doctor in order for them to get correct information on these practices and thought it was a doctor's right to hear their problems regarding T&CM even though they did not know the benefits of these practices. For instance, the patient disclosure of herbal use provides an opportunity for the doctors to advise them regarding the appropriate use of herbal remedies against conventional treatment in accordance with the severity of the conditions [22]. Besides, majority of the participants of current study had chronic diseases and had an opportunity to try any treatment which offers a cure from traditional and complementary medicine but it may put them at risk of side effects as well as unknown drug interaction due to lack of data regarding the safety of T&CM therapies [19].

Furthermore, this study found that the important factors motivating their disclosure of the benefits of T&CM to the medical doctor were their perception of the potential benefits of disclosure and various support from their spouse, family, and friends. Most of the participants thought that the main benefits of disclosing to the medical doctor were health benefits and medium of decision-making for continuation of these practices. Participants believed that they will get more information or better explanation form their doctor before they decided to continue to use T&CM. This finding was similar to the study done by Farooqui and colleagues, in which their participants believed that doctor might have some knowledge about traditional therapies and can better advise them about the T&CM [19]. This can explain that patients valued doctor's encouragement of patient involvement such as emphasizing patient's choice, shared responsibility, and giving reasons for advice in consultations [23]. Besides, the patient wants more information from their doctors and may want to actively participate in decision-making processes [24]. Furthermore, patients seem most likely to form a relationship with a doctor who meets their expectations or needs [25]. The lack of doctor's interest in this practice was among the reasons patients decided not to disclose their traditional and complementary medicine use [19]. This finding is also supported by another study where participants perceived doctor's limited knowledge on T&CM as reasons they might not be able to contribute useful information on T&CM use decision-making process [26]. Further, the lack of emphasis given on T&CM in the medical curriculum may indirectly contribute to the patient's perception of their doctor's having limited knowledge on T&CM and this in turn leads to their nondisclosure [19].

Besides, moral support from their spouse, family, and friends, who also use T&CM and perceived the benefits of these practices, encouraged them to tell their doctors pertaining to the issue of advantages and disadvantages of these practices. This is in line with the other study, where support from family (parent, spouse, and/or sibling) or other close persons (friend) was found to have a positive impact on their decision to confide in doctor [18].

However, fear of negative attitude and emotions from their doctors as well as fear of negative relationship development between doctor and patient was considered as their barrier to disclosure. This may lead to the implication of the possibility of T&CM-drug interaction especially to those who concurrently use T&CM and their Western medicine; hence any nondisclosure of T&CM may increase the risk of possible adverse reactions or interactions [27]. Furthermore, a study in UK found that people would be less likely to report adverse reactions to herbal compared to conventional medicine and this suggests that strategies need to be put in place to ensure communication of this type of information [28]. They concluded that the reasons for not reporting adverse herbal reactions were lack of realization of importance and fear of informing health professionals. The negative actions or responses by the doctor that include scolding or yelling would be worrisome to patients and could make them even more reluctant to disclose their traditional and complementary medicine [3, 19, 27, 29]. Similar to another study, they feared that disclosing T&CM to the doctors may lead to the reduction in the level of healthcare [19].

Besides, some of the participants in this study anticipated and were afraid that level of the trust between doctor and patient will be affected if they persistently disclose their use of these practices. They were afraid that their doctor will not believe them anymore as they tried to oppose their doctor's advice. The value of acceptance and nonjudgmental attitude by the doctor contributed to the willingness by the patient to disclose the use of T&CM and hence would help the patient overcome anticipated or previous negative interactions in a discussion about T&CM [30]. Therefore, safety and relationship between doctor and patient are likely to be better maintained if doctor is willing to enter into nonjudgmental dialogues with patients about T&CM and understand why this practice is meaningful to them and allow their patients to maintain personal control in decisions about treatment options [31].

Furthermore, doctor's interpersonal and communication characteristics of being involved, treating patients respectfully, listening attentively, respecting privacy, and leaving time for the patient are the critical component for disclosure. In addition to the processes by which patient-doctor relationships are developed and maintained, the studies suggested that depth of relationship, as a product of longitudinal care and consultation experiences, was important [24]. Besides, the literature on doctor-patient communication acknowledges the relevance of rapport in doctor-patient consultations. The ability of the doctor to communicate successfully largely depends on not only the doctor's clinical knowledge and technical skills, but also the nature of the rapport that is established between the doctor and the patient [32]. Furthermore, rapport building is characterized by a warm greeting, eye contact, a brief nonmedical interaction, or checking on an important life event of the patient. All these will go a long way to put the patient in a better and relaxing mood to open up to doctor in the consultation process [32]. This study highlighted the importance patient placed on not feeling hurried and their appreciation of doctors who had time [33]. However, some studies have explained that it is not the amount of time that is crucial in doctor-patient communication, but rather the way in which the available time is utilized [34, 35]. For some of the participants in this study, they thought that doctor who is respecting their privacy is related to their decision to disclose the use of T&CM. They believed that doctor will dealing their conversation correctly with confidentiality and keeping privacy. Professional secrecy is one of the most important ethical aspects of the doctor-patient relationship, as it establishes and ensures the trust that must exist between them [36].

In this study, satisfaction towards healthcare services represents the patient satisfaction level towards healthcare services which was rooted in the intrapersonal trust of the participants in the doctor. Most of the participants could utilize the trust built in the relationship to facilitate their satisfaction towards healthcare services in order to help them to be closer to the doctor, hence encouraging them to disclose the use of T&CM. No doubt, the patient satisfaction is one of the essential elements of quality care and an indication of good relationships between patients and doctors [37]. Trust is a vital element in the patient-doctor relationship; hence it enhances the disclosure process between them [38–40].

Gender differences were apparent in terms of influencing participant's decision to disclose the use of T&CM to the medical doctor. In this study, most of the participant's experiences were in their communication styles and health-seeking behaviour for disclosing their use of T&CM to the medical doctor. Gender is one of the factors that influence the communication [41]. In this study, we found that women are more open and receptive to health concern and expressing negative emotions and tend to share problems, whereas men always described themselves as healthy, tend to keep their own personal feelings to themselves, and tend to not share. This probably can be explained by the fact that women are completely at the mercy of their hormones [42]. Further, Feingold (1994) found that females were higher than males in extraversion, anxiety, trust, and especially tender-mindedness [43]. The masculine role interferes with and inhibits men's as well as women's emotional disclosure. Most studies assume that men who subscribe to the masculine role are characterized by a tendency to be less open and personally revealing about their emotions and feeling [44–46].

However, most of the participants preferred doctors of the same gender for disclosure. This can be explained by the fact that sex differences in self-disclosure may differ on the basis of sex target. Some studies found that sex differences in self-disclosure differ for same-sex and opposite-sex interactions [47]. Another study supported the statement that many women in the study were more willing to fully disclose their health issues to a female physician. They stated that they were often not comfortable discussing these sensitive issues [48]. There were gender differences in belief and health behaviour practices [49]. They found that male participants often defined being healthy as not seeking medical help. Therefore, attributing being healthy to not having a serious condition or to not needing to access healthcare services might reflect differing previous experiences of healthcare system utilization [49]. Hence they took telling their doctor about their use of traditional and complementary medicine for granted. Females on the other hand are more open and receptive to health concern especially on the efficacy or side effects of the traditional and complementary medicine.

While the current study has contributed to a better understanding and captured a wide range of views and experiences among primary care clinic attendees in Kuching Division, Sarawak, Malaysia, it has several limitations. There was the limit of the diversity of participants' experiences on disclosure especially when we use semistructured interviews as it prevented participants from expanding on their ideas. After all, due to its qualitative nature it may not be reflective of all patients with the disclosure of traditional and complementary medicine use to the medical doctor in the country.

## 5. Conclusion

In conclusion, this study identified participant's experience and views on the decision to disclose the use of T&CM to the medical doctor. These include their attitudes towards this practice, doctor's interpersonal and communication skills, intrapersonal trust regarding satisfaction towards healthcare services, and gender differences regarding disclosure. Thus, the doctor should consider gender differences in disclosure, their attitude towards T&CM use, and gained patient's trust in the delivery of healthcare services. A high quality of doctor's interpersonal and communication skills must be maintained for a good communication between doctor and their patient. However, more qualitative studies are needed alongside quantitative findings from healthcare provider's perspective in relation to the disclosure of T&CM context to help better understand the big picture of the patient-doctor relationship.

## Figures and Tables

**Table 1:** Sociodemographic and economic characteristic, health profile, and type of traditional and complementary medicine use of the participants (*N* = 10).

Characteristics	Frequency	Percentage
*Sociodemographic and economic characteristics*		
Age in years		
≤20	1	10.0
21–30	3	30.0
31–40	6	60.0
41–50	0	0.0
51–60	0	0.0
>60	0	0.0
Mean (SD) years	31.6 (6.6)	
Min–Max in years	20–40	
Mode in years	25	
Gender		
Female	7	70.0
Male	3	30.0
Ethnicity		
Malay	4	40.0
Chinese	1	10.0
Bidayuh	5	50.0
Religions		
Muslim	4	40.0
Christian	5	50.0
Buddhist	1	10.0
Education		
No formal education	0	0.0
Primary	1	10.0
Secondary	4	40.0
Tertiary	5	50.0
Marital status		
Married	8	80.0
Single	2	20.0
Occupation		
Unemployed	4	40.0
Private employee	3	30.0
Government employee	3	30.0
Household income per month		
≤MYR1000	5	50.0
MYR1001–2000	4	40.0
MYR2001–3000	1	10.0
MYR3001–4000	0	0.0
MYR4001–5000	0	0.0
>MYR5000	0	0.0
Mean (SD) MYR	1250.00 (754.62)	
Min–Max in MYR	500–2500	
Mode in MYR	500	

*HEALTH PROFILE*		
Type of chronic diseases^*∗*^		
Diabetes mellitus	5	50.0
Hypercholesterolemia	5	50.0
Hypertension	4	40.0
Asthma	3	30.0

*Type of T&CM use * ^*∗*^		
Supplement	10	100.0
Body massage	2	20.0
Malay herbs	1	10.0
Acupuncture	1	10.0

T&CM: traditional and complementary medicine; ^*∗*^multiple response.

## Appendix

Sample of interview questions

1. Tell me your experiences about the disclosure of the use of T&CM to doctor?
2. Probes:
  - How often do you tell your doctor?
  - What led you to disclose the use of T&CM doctor?
  - Are there any preferences of the doctor for you to tell your story?
  - Do you think the doctor should be told about the use of T&CM?
  - What will you get if you disclose the use of T&CM doctor?
  - What will happen if you do not disclose the use of T&CM to doctor?
  - How does your doctor react when you disclose the use of T&CM to doctor?
  - In your opinion, who will be more willing to tell the doctor about the use of T&CM? Why?
3. Tell me, what is your views on the use of traditional and complementary medicine?
4. Probe:
  - What led you to tell doctor on your perception of traditional and complementary medicine use?
  - Can you tell me, what are the important issues or components of disclosure of traditional and complementary medicine to your doctor?
  - How does your doctor react to giving their feedback on your perception of traditional and complementary medicine used?
  - Is there anyone influence your decision to disclose the use of traditional and complementary medicine to your doctor?
  - Do you have any knowledge about the selection of the right T&CM?
  - Have you ever tell your doctor on how to select or choose the right traditional and complementary medicine?
5. Are you satisfied with the service you received at this clinic?
6. Probes:
  - How is doctor serve you? Why are you satisfy/dissatisfied with their service?
  - Are the services given by doctors encourage you to disclose the use of T&CM to doctor?
  - Financially, is it prevent you to get a better service in here?
  - Does it encourage you to disclose the use of T&CM to doctor?
7. How is your relationship with your doctor so far?
8. Probes:
  - Are these relationships can encourage you to disclose the use of T&CM to the doctor?
  - How your doctor act during conversation?
  - Are doctors readily available here for you to get health care services?
  - Will it encourage you to disclose the use of T&CM to doctor?
  - In your opinion, how to encourage the positive relationship between you and doctor?

## Abbreviations

MYR: Malaysian Ringgit
MOH: Ministry of Health
SD: Standard Deviation
T&CM: Traditional and complementary medicine
WHO: World Health Organization.

## Additional Points

*Availability of Data and Materials*. The datasets used and/or analyzed during the current study have been kept confidential and are not available publicly.
